# α_v_β_3_ integrin-targeted magnetic resonance imaging in a pancreatic cancer mouse model using RGD-modified liposomes encapsulated with Fe-deferoxamine

**DOI:** 10.1371/journal.pone.0310984

**Published:** 2024-09-24

**Authors:** Mitsuyoshi Yoshimoto, Takuya Hayakawa, Masayuki Yamaguchi, Sadaaki Kimura, Hirofumi Fujii

**Affiliations:** Division of Functional Imaging, Exploratory Oncology Research & Clinical Trial Center, National Cancer Center, Kashiwa, Chiba, Japan; Queen’s University Belfast, UNITED KINGDOM OF GREAT BRITAIN AND NORTHERN IRELAND

## Abstract

Magnetic resonance (MR) imaging is a powerful imaging modality for obtaining anatomical information with high spatial and temporal resolution. In the drug delivery system (DDS) framework, nanoparticles such as liposomes are potential candidates for MR imaging. We validated that RGD peptides are possible targeting molecules for pancreatic cancer with α_v_β_3_ integrin expression. This study aimed to evaluate RGD-modified liposomes loaded with ferrioxamine B for pancreatic cancer imaging. We synthesized four types of RGD-modified liposomes encapsulated with ferrioxamine B (SH-, H-, M-, and L-RGD-liposomes). The binding affinity of RGD-modified liposomes was evaluated in a competitive inhibition study using ^125^I-echistatin. To investigate the pharmacokinetics of RGD-modified liposomes, a biodistribution study using RGD-liposomes labeled with ^111^In was carried out in a human pancreatic cancer PANC-1 xenograft mouse model. Finally, MR was performed using ferrioxamine-B-loaded liposomes. RGD-liposomes inhibited the binding of ^125^I-echistatin to RGD. The biodistribution study revealed that ^111^In-RGD-liposomes accumulated significantly in the liver and spleen. Among the ^111^In-RGD-liposomes, ^111^In-H-RGD-liposomes showed the highest tumor-to-normal tissue ratio. In the MR study, H-RGD-liposomes loaded with ferrioxamine B showed higher tumor-to-muscle signal ratios than RKG-liposomes loaded with ferrioxamine B (control). We successfully synthesized RGD-liposomes that can target α_v_β_3_ integrin.

## Introduction

Magnetic resonance (MR) imaging is a highly desirable modality in molecular imaging. MR imaging has superior spatial and temporal resolution than positron emission tomography (PET) and single-photon emission computed tomography (SPECT). In addition, contrast-enhanced MR is superior or even at par with computed tomography (CT) for pancreatic cancer imaging [1–3]. However, the lower sensitivity of MR to contrast agents compared to PET/SPECT is a barrier to the successful development of molecular probes for MR with targeting abilities.

Using nanoparticles such as liposomes and micelles is a possible strategy for developing molecular probes for MR imaging [4, 5]. In recent decades, nanoparticles have been extensively used as drug carriers to improve pharmacokinetics or deliver hydrophobic drugs [6, 7]. Moreover, modifying the surface of nanoparticles with peptides or antibodies allows for targeting lesion sites, blood vessels, or tumors. Zhang et al. reported that gadolinium diethylenetriamine pentaacetic acid (Gd-DTPA) liposomes modified with anti-CD105 antibodies could detect tumor angiogenesis [8]. Jacobin-Valat et al. synthesized nanoparticles functionalized with anti-platelet antibodies for the MR of atherosclerotic plaques and demonstrated that these nanoparticles bound to atheroma plaques [9]. Thus, cell/tissue-specific MR imaging could be enhanced using nanoparticles conjugated with targeting molecules, such as peptides and antibodies.

Integrin α_v_β_3_ is overexpressed in endothelial cells and various tumor cells, including pancreatic cancer [10–13] and in a pancreatic ductal carcinogenesis model [14]. In addition, we previously reported that SPECT using ^111^In-1,4,7,10-tetraazacylododecane-N,N′,N″,N′′′-tetraacetic acid-cyclo-(Arg-Gly-Asp-D-Phe-Lys) (^111^In-DOTA-c(RGDfK)) successfully detected pancreatic cancers in a hamster carcinogenesis model [15]. Therefore, α_v_β_3_ integrin is a potential target for drug development in pancreatic cancer and brain tumors.

The purpose of this study is to evaluate RGD-modified liposomes loaded with ferrioxamine B for pancreatic cancer imaging. We synthesized RGD-modified liposomes loaded with Fe-deferoxamine (ferrioxamine B) for MR imaging. We believe that combining the targeting capabilities of the RGD peptide and drug delivery system (DDS) of liposomes could improve MR imaging detection of intractable tumors, such as pancreatic cancer and brain tumors.

## Methods

### Thiolation of c(RGDfK)

**Fig 1:**
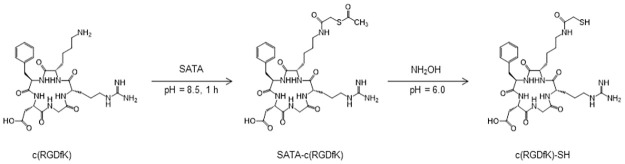
Synthetic scheme of c(RGDfK)-SH. To introduce thiol into c(RGDfK), SATA was conjugated to c(RGDfK). Subsequently, the sulfhydryl group was deprotected using hydroxylamine.

Thiolation of c(RGDfK) was conducted as described in a previous report (Fig 1) [16]. As previously reported, both c(RGDfK) and c(KGfDR) as a control peptide (RKG) were synthesized [17]. Briefly, 50 μmol of c(RDGfK) was dissolved in 10 mL of 0.5 M borate buffer (pH 8.5). Next, 70 μmol of *N*-succinimdyl *S*-acetylthioacetate (SATA) in dimethyl sulfoxide (DMSO) (1.2 mL) was added. The solution was stirred at room temperature (RT) for 1 h, after which 1 mL of 2% trifluoroacetic acid (TFA) in H_2_O was added to terminate the reaction. The solvent was then removed *in vacuo* to yield SATA-c(RGDfK) as a white solid (21 mg). Subsequently, 2 mL of 0.5 M NH_2_OH·HCl was added to 50 μmol of SATA-c(RGDfK) in H_2_O (5 mL), and the pH was adjusted to pH 6.0, using 0.5 M NaOH. The resulting solution was stirred at RT for 1 h and then purified using high pressure liquid chromatography (HPLC) on a Cosmosil 5C18 AR-II (10 × 250 mm; Nacalai, Kyoto, Japan). It was eluted using a gradient of solvent A (0.1% TFA in water) and solvent B (0.1% TFA in CH_3_CN), ramping from 15% to 35% solvent A over 20 min while maintaining a flow rate of 5 mL/min. Finally, the resulting solution was lyophilized to yield c(RGDfK)-SH (19 mg, yield: 56.4%).

### Synthesis of ferrioxamine B (a complex of Fe and deferoxamine)

To obtain ferrioxamine B, 70 mM FeCl_3_ in water (100 μL) was added to 7.7 μmol deferoxamine mesylate (Sigma-Aldrich). The solution was then purified using a Sep-Pak C18 cartridge (Waters, Milford, MA, USA). The solution was passed through the cartridge, which was then washed with water. Ferrioxamine B was eluted using 50% methanol and the eluent was lyophilized.

### Synthesis of liposomes encapsulated with ferrioxamine B or deferoxamine

Liposomes composed of 1,2-distearoyl-sn-glycero-3-phosphocholine (DSPC), cholesterol, N-(carbonyl-methoxypolyethyleneglycol 2000)-1,2-distearoyl-sn-glycero-3-phosphoethanolamine (mPEG-DSPE), and maleimide-mPEG-DSPE were prepared using a thin-film hydration method [18, 19].

Briefly, the lipids were dissolved in chloroform and the solvent was evaporated. The molar ratio of the lipids was as follows: DSPC:cholesterol:mPEG-DSPE = 48.9:44.4:6.67. The amount of RGD modification was regulated by changing the ratio of maleimide-mPEG-DSPE to the total mPEG-DSPE (Table 1). The dried lipid film was hydrated in 100 mM ferrioxamine B for MR imaging or 6.4 mM deferoxamine for ^111^In labeling and dissolved in 30 mM HEPES/5% mannitol buffer (pH 7.4) at 60°C. The lipid dispersion was extruded 15 times through layered 0.2-μm polycarbonate filters to prepare 100-nm-diameter liposomes. The liposomes were then purified via Sephadex G-50 column chromatography (GE Healthcare Japan Ltd., Tokyo, Japan) to remove non-encapsulated ferrioxamine B and deferoxamine. The phospholipid concentration of liposomes was measured using a commercially available assay kit (Phospholipid C-Test Wako; Wako Pure Chemicals, Osaka, Japan). The amount of Fe was measured using liquid chromatography–mass spectrometry (LC/MS) analysis. LC/MS analysis was performed on a Prominence UFLC system (Shimadzu, Kyoto, Japan)-API 3200 (AB SCIEX, Toronto, Canada). The amount of Fe encapsulated in the liposomes was 15.18 ± 1.23 μg/μmol lipid.

**Table 1:** Lipid composition of the liposomes.

	DSPC	Cholesterol	mPEG-DSPE	Maleimide-mPEG-DSPE
SH-RGD-liposome	48.9%	44.4%	3.33%	3.33%
H-RGD-liposome RKG-liposome			6.33%	0.33%
M-RGD-liposome			6.50%	0.17%
L-RGD-liposome			6.60%	0.07%
NT-liposome			6.67%	0.00%

### Coupling of RGD or RKG peptides to liposomes

**Fig 2:**
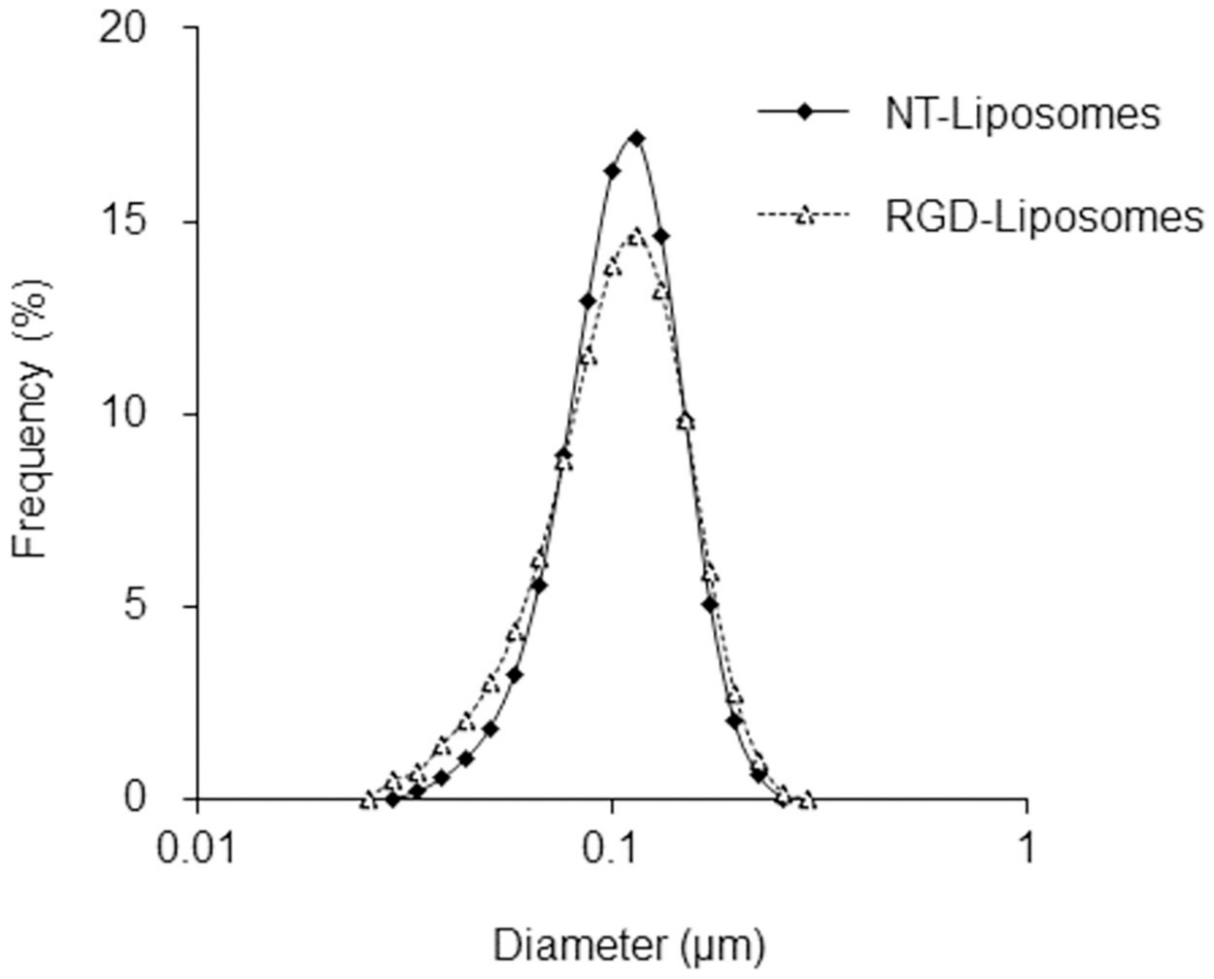
Particle size distribution curves of RGD-liposomes and NT-liposomes.

To prepare c(RGDfK) or c(KGfDR)-modified liposomes (generating RGD-liposomes and RKG-liposomes, respectively), c(RGDfK) or c(KGfDR) was conjugated to the liposomes via sulfhydryl-maleimide coupling [16]. Briefly, thiolated peptides (74 nmol) were added to the liposome solutions (6.7 nmol as maleimide). The ratios of maleimide-mPEG-DSPE were 3.33% for SH-RGD-liposomes, 0.33% for H-RGD-liposomes, 0.17% for M-RGD-liposomes, 0.07% for L-RGD-liposomes, and 0.33% for RKG-liposomes (Table 1). The pH levels of the solutions were adjusted to 7.5 with 0.5 M borate buffer (pH 8.5) and stirred at RT for 2 h. Uncoupled peptides were separated from the liposomes via ultracentrifugation (100,000 rpm, 20°C, 20 min). The liposomes were then washed twice with phosphate-buffered saline. The particle sizes of the RGD/RKG-liposomes and NT-liposomes were 103.7 ± 3.9 nm and 103.2 ± 1.5 nm, respectively (Fig 2).

### Cell culture and animal model

The human pancreatic ductal carcinoma cell line (PANC-1; American Type Culture Collection, Manassas, VA, USA) was used in this study. PANC-1 cells were cultured in Dulbecco’s modified Eagle’s medium (4.5 g/L glucose; Invitrogen, Carlsbad, CA, USA) supplemented with 10% fetal bovine serum and maintained at 37°C in a humidified atmosphere of 5% CO_2_ in air. Animal studies were conducted using SCID mice (CB17/ICr-scid/scid Jcl, 5–7 weeks old; CLEA Japan) bearing PANC-1 cells. Mice were xenografted subcutaneously with 5 × 10^6^ PANC-1 cells into the right dorsum to establish the animal model. All experiments were conducted in accordance with the ARRIVE guidelines (https://arriveguidelines.org). The study protocol was received approval from the Committee for Ethics of Animal Experimentation at the National Cancer Center (K15-011). Animal experiments adhered to the committee’s Guidelines for the Care and Use of Experimental Animals. All invasive procedures were performed under isoflurane anesthesia to alleviate suffering.

### Cell-binding assay for liposomes

The binding affinities of the liposomes to α_v_β_3_ integrin were assessed using a competitive cell-binding assay [16]. ^125^I-echistatin was used as a radioligand for α_v_β_3_ integrin. Initially, 2 × 10^5^ PANC-1 cells were incubated with 74 kBq ^125^I-echistatin and liposomes in cell-binding buffer (25 mM Tris-HCl, 150 mM NaCl, 1 mM CaCl_2_, 0.5 mM MgCl_2_, 1 mM MnCl_2_, and 0.1% polyethyleneimine; pH 7.4). The lipid concentration of the liposomes ranged from 0.01 μM to 1 mM in this experiment. After 2 h of incubation, the cells were filtered through GF/B filter papers using a Brandel cell harvester (Brandel, Hertfordshire, UK), followed by five washes with 3 mL of cell-binding buffer. After the filters were dried, their radioactivity was measured using an automated gamma counter (2480 WIZARD, Perkin Elmer Japan, Kanagawa, Japan). The IC_50_ (50% inhibitory concentration) values were obtained by fitting the data using nonlinear regression with GraphPad Prism 9 (GraphPad Software, San Diego, CA, USA).

### ^111^In labeling of liposomes

Liposomes were radiolabeled using a remote loading method [18, 20]. To prepare ^111^In-oxine complexes, ^111^In-InCl_3_ was mixed with 0.5 mM oxine (8-quinolinol) in EtOH and incubated in 0.5 M acetate buffer (pH 6.0) for 30 min at 37°C. Deferoxamine-encapsulated liposomes were then incubated with the ^111^In-oxine solution for 30 min at 37°C. Excess ^111^In-oxine was removed via ultracentrifugation (100,000 rpm, 20°C, 20 min). Afterward, the liposomes were washed twice with saline.

### Biodistribution studies

The biodistribution of RGD-modified liposomes loaded with ^111^In was evaluated in a PANC-1 xenograft model. Mice were injected with 37 kBq of ^111^In-liposomes via the tail vein (n = 4 for each time point). At designated time intervals, mice were euthanized by cervical dislocation under deep isoflurane anesthesia, and their organs were dissected. The tissues were weighed, and their radioactivity was measured using an automated gamma counter. Data were calculated and reported as the percentage of the injected dose per gram of tissue.

### MR imaging with ferrioxamine B-loaded liposomes

MR imaging using H-RGD- and RKG-liposomes loaded with ferrioxamine B was performed in a PANC-1 xenograft model. First, ferrioxamine B-loaded liposomes (0.42–0.48 mg Fe) were injected via the tail vein of the mice. The mice were then anesthetized during MR imaging, which was performed using a 9.4-Tesla animal scanner (Biospec 94/20 USR; Bruker BioSpin, Ettlingen, Germany) equipped with an 8-channel multi-array coil (Mouse Body Array Coil, Bruker BioSpin, 72 mm ID) at 4 and 24 h post-injection (pi). T_1_-weighted images were acquired with a fast spin-echo pulse sequence using the following parameters: repetition time, 476 ms; echo time, 8.7 ms; flip angle, 90°; in-plane resolution, 156 μm × 156 μm; slice thickness, 1 mm; and number of excitations, 4.

To evaluate the contrast enhancement in the tumor after administering ferrioxamine B-encapsulated liposomes, the tumor-to-muscle signal ratios were calculated using ImageJ software (available from http://imagej.nih.gov/ij, Bethesda, MD) as follows. On the slice section containing the maximum diameter of the tumor, regions of interest (ROIs) were manually placed on the tumor and paraspinal muscle. Subsequently, the average pixel values of the individual ROIs were recorded as signal intensities. The relative intensity of the tumor was calculated as the signal intensity of the ROI in the tumor divided by the signal intensity of the ROI in the paraspinal muscle.

The T_1_ relaxivity values of the ferrioxamine B solution and ferrioxamine B-loaded liposome suspensions were measured using the same MR imaging scanner described above. MR images of the phantoms containing either ferrioxamine B solution with various iron concentrations (0–1.2 mM, six steps) or ferrioxamine B-loaded liposome suspensions with equivalent iron concentrations were acquired with a fast spin-echo pulse sequence (RARE T1 T2 map; Bruker Biospin) using the following parameters: repetition times, 200–5500 ms (in six steps); echo times, 10–90 ms (in five steps); flip angles, 90°; in-plane resolution, 156 × 156 μm; slice thickness, 1 mm; and number of excitations, 1. T_1_ maps were generated using software equipped with the MR imaging scanner. The inverses of the T_1_ relaxation times of the phantoms were plotted as a function of the iron concentration. After applying least-square fitting to the plots, we recorded the slope values as the T_1_ relaxivity values of the ferrioxamine B solution and ferrioxamine B-loaded liposome suspensions.

### Statistical analysis

Data were analyzed using GraphPad Prism 9 (GraphPad Software). Differences between groups were analyzed using a two-way analysis of variance, followed by Dunnett’s test or Sidak test for multiple comparisons. Statistical significance was set at *p* < 0.05 (*p < 0.05, **p < 0.01, ***p < 0.001, and ****p < 0.0001). Data are presented as mean ± standard deviation.

## Results

### Specific binding of RGD-liposomes to α_v_β_3_ integrin

**Fig 3:**
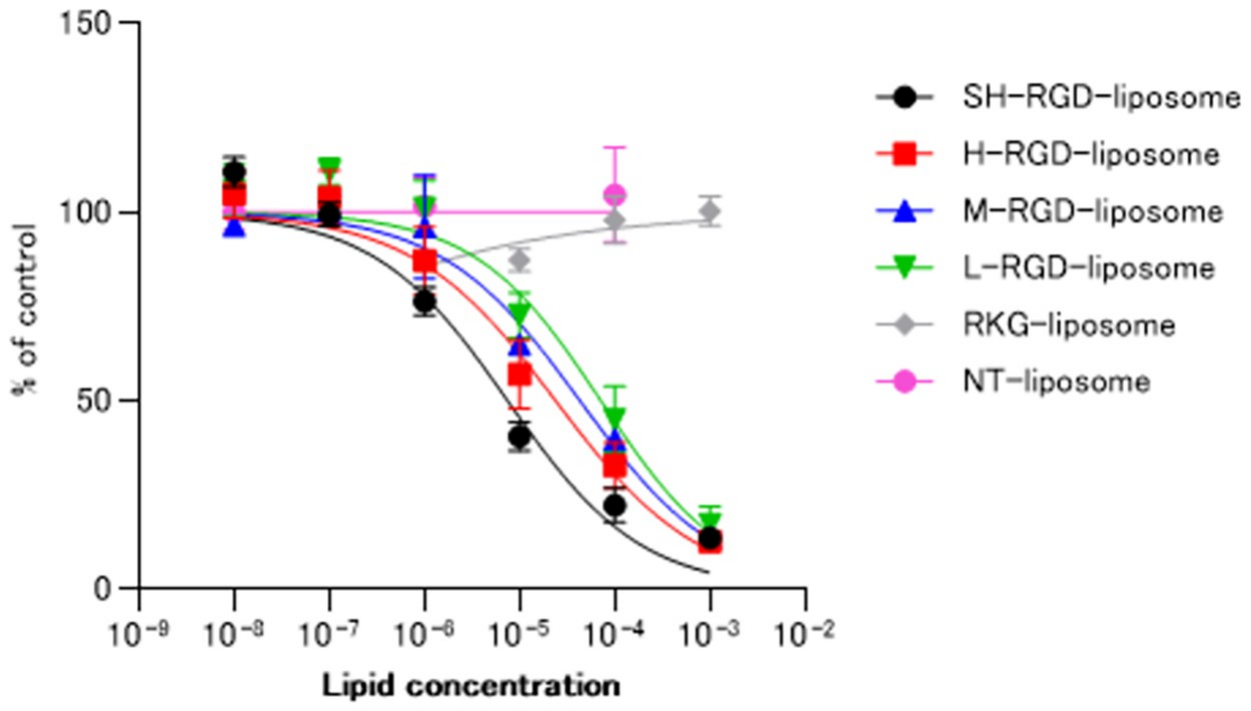
Inhibition of ^125^I-echistatin binding to α_v_β_3_ integrin on PANC-1 cells by RGD-, RKG-, and NT-liposomes (n = 3). The x-axis represents the concentration of the liposomal membrane lipid. The RGD-liposomes dose-dependently inhibited the binding of ^125^I-echistatin to PANC-1 cells in a dose-dependent manner. However, no inhibition was observed with RKG- or NT-liposomes.

To estimate the binding ability of RGD-liposomes to α_v_β_3_ integrin, an inhibition study of ^125^I-echistatin was carried out using PANC-1 cells that have a high expression of α_v_β_3_ integrin. The results showed that RGD-liposomes dose-dependently inhibited the binding of ^125^I-echistatin to PANC-1 cells (Fig 3, S1 in S1 File). In addition, the binding of statins was strongly inhibited depending on the amount of RGD modification on liposomes. The IC_50_ values were 6.67 ± 2.11 μM, 20.09 ± 5.86 μM, 42.66 ± 10.92 μM, and 70.63 ± 18.59 μM for SH-RGD-liposomes, H-RGD-liposomes, M-RGD-liposomes, and L-RGD-liposomes, respectively. In contrast, the RKG-modified liposomes and NT-liposomes did not inhibit the binding of ^125^I-echistatin to PANC-1 cells.

### Biodistribution of ^111^In-RGD- and ^111^In-NT-liposomes

**Fig 4:**
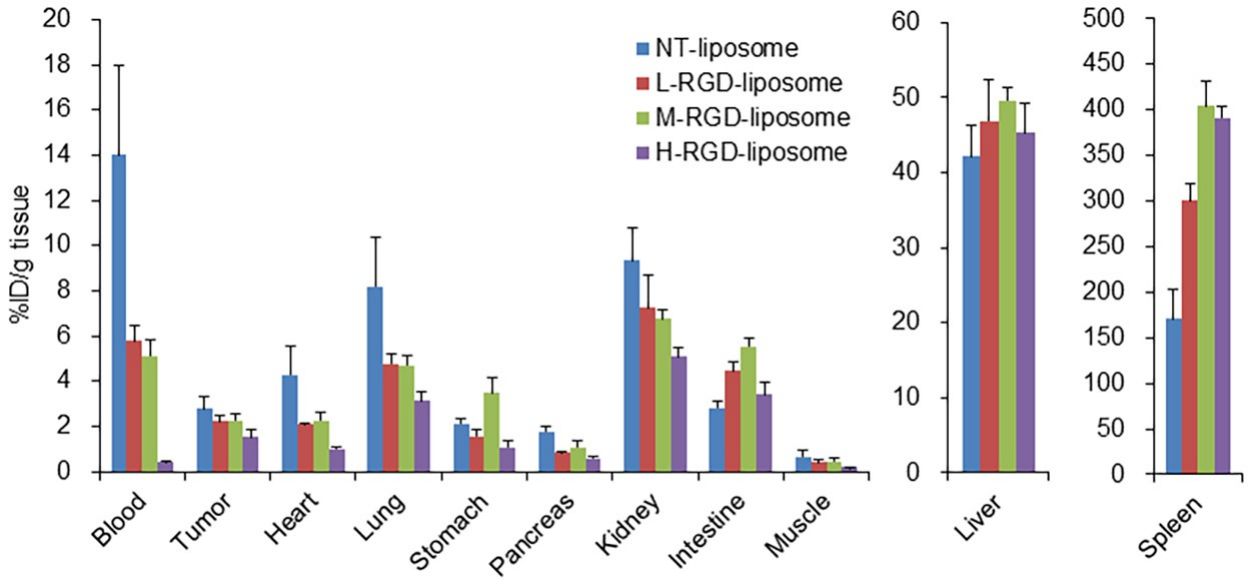
Biodistribution of ^111^In-RGD-liposomes and ^111^In-NT-liposome in PANC-1 xenograft-bearing nude mice at 24 h post-injection (n = 3–4). Results are expressed as the percentage of the ID/g tissue. The ^111^In-NT-liposome showed the highest accumulation in both tumor and blood. Among the ^111^In-RGD-liposomes, the ^111^In-H-RGD-liposome showed the lowest accumulation in these tissues. Accumulation in the spleen varied with the degree of RGD peptide modification on the liposomes.

**Fig 5:**
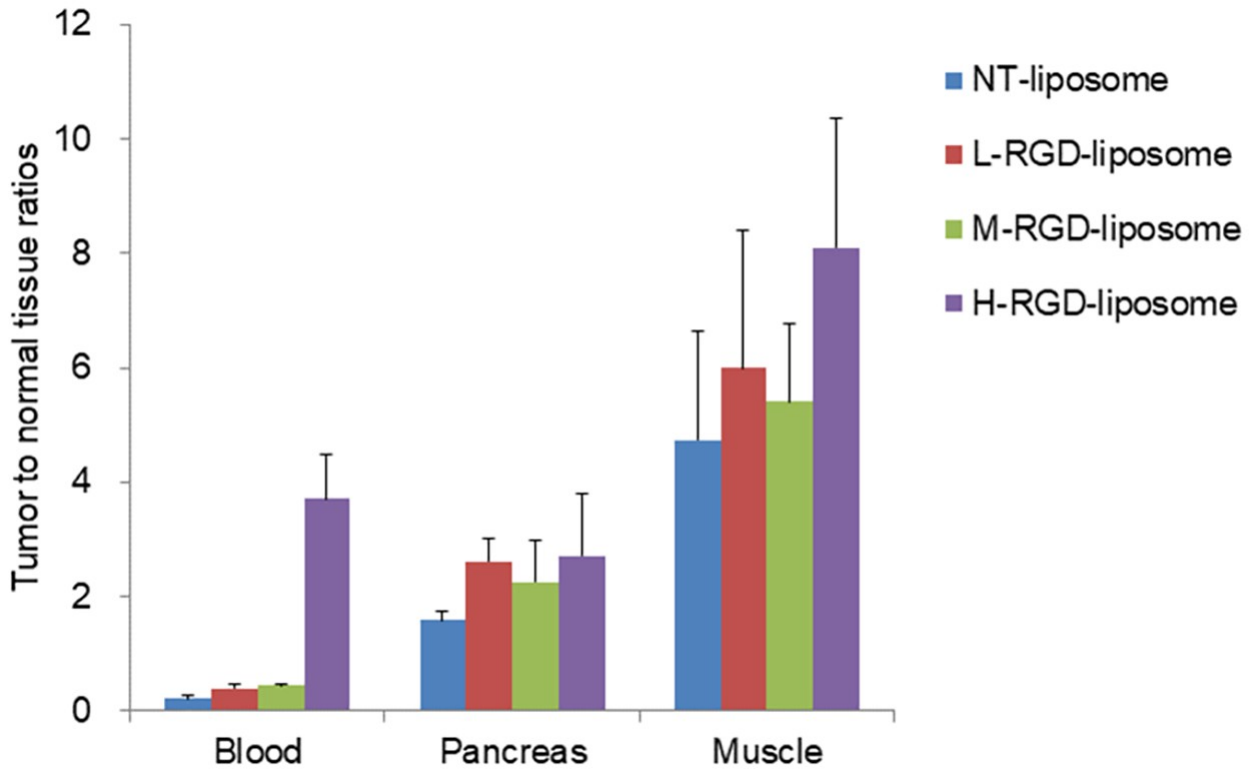
Tumor-to-blood, -pancreas, and -muscle ratios of ^111^In-RGD-liposomes and ^111^In-NT-liposomes in PANC-1 xenograft-bearing nude mice at 24 h post-injection (n = 4). Tumor to normal tissue ratios were calculated from the biodistribution data.

Biodistribution studies indicated the rapid clearance of ^111^In-SH-RGD-liposomes from blood (0.41 ± 0.10% ID/g at 4 h) and low tumor uptake (0.44 ± 0.12% ID/g at 4 h). A significant accumulation of radioactivity in the spleen was observed with increasing amounts of RGD modification: 170.42 ± 33.28% ID/g for ^111^In-NT-liposomes, 300.29 ± 18.57% ID/g for ^111^In-L-RGD-liposomes, 403.92 ± 27.69% ID/g for ^111^In-M-RGD-liposomes, and 391.31 ±12.72% ID/g for ^111^In-H-RGD-liposome at 24 h (Fig 4, S2 in S1 File). While ^111^In-NT-liposomes exhibited the highest tumor uptake (2.80 ± 0.53% ID/g) at 24 h, the RGD-modified liposomes showed the lowest tumor uptake (2.21 ± 0.29 for ^111^In-L-RGD-liposomes, 2.27 ± 0.30 for ^111^In-M-RGD-liposomes, 1.52 ± 0.35 for ^111^In-H-RGD-liposomes % ID/g). However, the tumor-to-blood (T/B) and tumor-to-muscle (T/M) ratios for the ^111^In-H-RGD-modified liposomes were the highest among all the liposomes used in this study (3.71 ± 0.79 and 8.10 ± 2.25, respectively; Fig 5, S3 in S1 File).

### MR imaging

**Fig 6:**
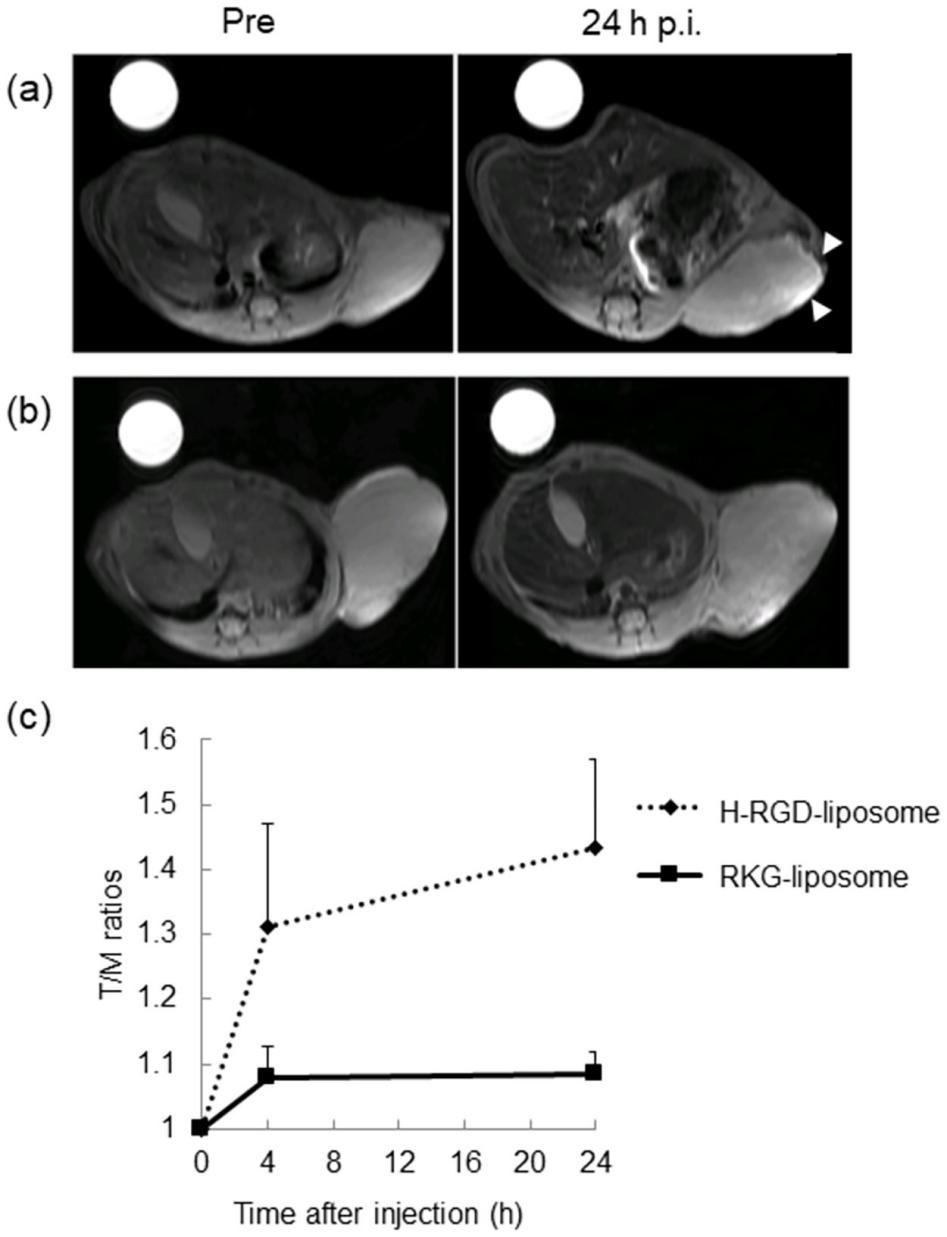
MR imaging (9.4 T) of PANC-1 xenograft-bearing scid mice. (a) RARE T_1_ MR images after treatment with H-RGD-liposomes loaded with ferrioxamine B. Open arrowheads indicate enhanced areas. (b) RARE T_1_ MR images after treatment with RKG-liposomes loaded with ferrioxamine B. (c) Tumor-to-muscle signal (T/M) ratios for H-RGD- and RKG-liposomes loaded with ferrioxamine B (n = 3).

Fig 6a and 6b show representative MR images of the PANC-1 xenograft after administration of either H-RGD-liposomes loaded with ferrioxamine B or RKG-liposomes loaded with ferrioxamine B. Before administration, the PANC-1 xenograft exhibited an equivalent signal intensity compared to the muscle, thereby giving a tumor-muscle ratio of approximately 1.0. After administering H-RGD-liposomes loaded with ferrioxamine B, the tumor-muscle ratio reached 1.31 ± 0.16 and 1.39 ± 014 at 4 and 24 h, respectively. In contrast, the tumor-muscle ratio only increased slightly to 1.08 ± 0.05 at 4 h after administration of RKG-liposomes loaded with ferrioxamine B. However, it remained unchanged (1.08 ± 0.04) at 24 h (Fig 6c, S4 in S1 File). Visually, H-RGD-liposomes loaded with ferrioxamine B enhanced the signals, especially in the tumor margin, whereas RKG-liposomes loaded with ferrioxamine B did not. The T_1_ relaxivity values of the ferrioxamine B solution and ferrioxamine B-loaded liposome suspension were 2.6 and 1.9 mM^-1^ sec^-1^, respectively.

## Discussion

Liposomes that can encapsulate drugs are potential candidates for drug delivery systems. Liposomal doxorubicin and irinotecan have already been used for cancer treatment [21, 22]. In addition, liposomes can be modified with antibodies and peptides to enhance their targeting ability [23–26]. In this study, we successfully synthesized RGD-modified liposomes loaded with ferrioxamine B to improve imaging contrast of pancreatic cancers in MR imaging.

The ratio of maleimide-mPEG-DSPE was adjusted to regulate the amount of RGD modification. Although we did not quantitatively determine the exact amount of modified RGD, the increased inhibition of ^125^I-echistatin binding by the H-RGD-liposomes compared to the M- or L-RGD-liposomes suggests effective control over RGD modification. We achieved this by varying the amount of maleimide-mPEG-DSPE. Conversely, Kluza et al. controlled the modification of RGD and anginex on liposomes by altering the peptide amounts [19]. They demonstrated that liposomes heavily modified with peptides were more readily taken up by human umbilical vein endothelial cells. This suggests that sulfhydryl-maleimide coupling is a suitable method for regulating peptide or antibody conjugation to nanoparticles.

Inhibition experiments against ^125^I-echistatin also showed an increased binding affinity with increasing c(RGDfK) concentration. In contrast, the binding affinity of RKG-liposomes and unmodified liposomes was not observed in this study. These results suggest that the RGD-liposomes specifically bind to α_v_β_3_ integrin, and the binding capacity is dependent on the amount of modification. Previous studies have shown that the multimerization of RGD peptides enhances their binding affinity [27, 28]. Thus, increasing the modification of c(RGDfK) on the small surface of the liposomes would result in the same effect as multimerization, leading to enhanced binding affinity.

The biodistribution of liposomes encapsulated with ^111^In was evaluated in our current model. The H-RGD-liposomes showed the highest T/N ratio, although their tumor uptake was lower than the other liposomes. The other liposomes showed high radioactivity in the blood, which contributes to tumor uptake, indicating nonspecific accumulation in tumors. Therefore, H-RGD-liposomes are possible candidates for α_v_β_3_ integrin-targeting MR imaging agents.

Surprisingly, the biodistribution of liposomes with or without RGD peptides differed considerably. RGD conjugation did not enhance tumor targeting of the liposomes. This may be ascribed to increased spleen uptake, resulting in decreased blood radioactivity. The blood clearance of the ^111^In-NT-liposomes was very slow (14% at 24 h), leading to elevated tumor uptake. Conversely, RGD-liposomes were rapidly eliminated from the blood, depending on the extent of RGD modification. This rapid elimination could be due to their uptake in the spleen, as the splenic uptake of ^111^In-M- or H-RGD-liposomes was more than twice that of ^111^In-NT-liposomes. In ^111^In-NT-liposomes, the PEG chains form a hydration field and inhibit the adsorption of opsonic molecules through steric hindrance, preventing their capture by the reticuloendothelial system [29–32].

However, in ^111^In-RGD-liposomes, it is believed that the c(RGDfK) moieties at the tips of the PEG molecules interfere with the formation of this hydration field. There have been some reports of RGD-modified liposomes that encapsulate anti-cancer drugs and small interfering RNA [33–36]. However, these studies did not evaluate the pharmacokinetics of these drugs. Li et al. reported that the RGD10 modification of liposomes enhanced their blood clearance [37]. In their study, the amount of RGD modification was approximately 0.15% of the total lipids, which was equivalent to that in the M-RGD-liposomes in the current study. This study strongly supports our result that RGD modification hampers the water layer by PEG.

Encapsulated H-RGD-liposomes showed improved contrast enhancement compared to RKG-liposomes, indicating that RGD modification could enhance the tumor-targeting ability of liposomes. Unfortunately, the T_1_ relaxation time shortening effect was limited, although we successfully entrapped a high concentration of ferrioxamine B into the liposomes. We speculated that this could be because the encapsulation of ferrioxamine B restricts the interaction between ferrioxamine B and the protons of water molecules in small spaces such as liposomes. It is believed that the T1 relaxivities of the ferrioxamine B-encapsulated liposomes are lower than those of clinically used Gd-DTPA (R1 = 7.7 mM^-1^ sec^-1^), which could lead to less distinct enhancement on the MR images.

To enhance the relaxivity of liposomes, one approach could be the encapsulation of Gd-DTPA. Alternatively, utilizing magnetic materials that exhibit strong T2 relaxation effects could be effective. This effect results not only from the material’s magnetic susceptibility but also from its interaction with the protons of water molecules. For instance, the utilization of superparamagnetic iron with strong T_2_ relaxivities (R2 = 250 mM^-1^ sec^-1^) could overcome this issue [38]. Recently, superparamagnetic liposomes encapsulated in γ-Fe_2_O_3_ have been used as MR contrast agents [4, 39, 40].

In tumor imaging and therapy, pharmacokinetics and targeting ability are pertinent for drug development. In this study, we successfully conferred targeting capability to liposomes through RGD conjugation. However, we found that RGD reduces the effectiveness of PEG on the liposome surface. To improve the pharmacokinetics of RGD-liposomes, it is necessary to optimize the RGD concentration on the liposome surface to avoid disrupting the formation of the hydration field.

## Conclusions

We successfully developed RGD-modified liposomes that could target pancreatic cancer cells via the α_v_β_3_ integrin. Unfortunately, ^111^In-RGD-liposomes exhibited increased uptake in the spleen and low tumor uptake, leading to limited tumor contrast enhancement. To improve these pharmacokinetics, are necessary, including optimizing RGD modification and exploring the use of ferromagnet materials like superparamagnetic iron to enhance contrast. Overcoming these challenges could remarkably advance the use of RGD-liposomes in diagnosing and treating pancreatic cancer.

## Supporting information

S1 File (XLSX)

## References

[1] Nishiharu T, Yamashita Y, Abe Y, Mitsuzaki K, Tsuchigame T, Nakayama Y, et al. Local extension of pancreatic carcinoma: assessment with thin-section helical CT versus with breath-hold fast MR imaging—ROC analysis. Radiology. 1999;212: 445–452. doi: 10.1148/radiology.212.2.r99au09445 . 10429702

[2] Schima W, Függer R, Schober E, Oettl C, Wamser P, Grabenwöger F, et al. Diagnosis and staging of pancreatic cancer: comparison of mangafodipir trisodium-enhanced MR imaging and contrast-enhanced helical hydro-CT. AJR Am J Roentgenol. 2002;179: 717–724. doi: 10.2214/ajr.179.3.1790717 . 12185052

[3] Trede M, Rumstadt B, Wendl K, Gaa J, Tesdal K, Lehmann KJ, et al. Ultrafast magnetic resonance imaging improves the staging of pancreatic tumors. Ann Surg. 1997;226: 393–405; discussion 405; discussion. [Epub 1997/11/14]. doi: 10.1097/00000658-199710000-00001 . 9351708 PMC1191049

[4] Fortin-Ripoche JP, Martina MS, Gazeau F, Ménager C, Wilhelm C, Bacri JC, et al. Magnetic targeting of magnetoliposomes to solid tumors with MR imaging monitoring in mice: feasibility. Radiology. 2006;239: 415–424. Epub 2006/03/22. doi: 10.1148/radiol.2392042110 . 16549622

[5] Martina MS, Fortin JP, Ménager C, Clément O, Barratt G, Grabielle-Madelmont C, et al. Generation of superparamagnetic liposomes revealed as highly efficient MRI contrast agents for in vivo imaging. J Am Chem Soc. 2005;127: 10676–10685. doi: 10.1021/ja0516460 . 16045355

[6] Allen TM. Long-circulating (sterically stabilized) liposomes for targeted drug delivery. Trends Pharmacol Sci. 1994;15: 215–220. doi: 10.1016/0165-6147(94)90314-x . 7940982

[7] Lukyanov AN, Torchilin VP. Micelles from lipid derivatives of water-soluble polymers as delivery systems for poorly soluble drugs. Adv Drug Deliv Rev. 2004;56: 1273–1289. doi: 10.1016/j.addr.2003.12.004 . 15109769

[8] Zhang D, Feng XY, Henning TD, Wen L, Lu WY, Pan H, et al. MR imaging of tumor angiogenesis using sterically stabilized Gd-DTPA liposomes targeted to CD105. Eur J Radiol. 2009;70: 180–189. doi: 10.1016/j.ejrad.2008.04.022 . 18541399

[9] Jacobin-Valat MJ, Laroche-Traineau J, Larivière M, Mornet S, Sanchez S, Biran M, et al. Nanoparticles functionalised with an anti-platelet human antibody for in vivo detection of atherosclerotic plaque by magnetic resonance imaging. Nanomedicine. 2015;11: 927–937. doi: 10.1016/j.nano.2014.12.006 . 25684334

[10] Brooks PC, Clark RA, Cheresh DA. Requirement of vascular integrin alpha v beta 3 for angiogenesis. Science. 1994;264: 569–571. doi: 10.1126/science.7512751 . 7512751

[11] Hamdan S, Verbeke CS, Fox N, Booth J, Bottley G, Pandha HS, et al. The roles of cell surface attachment molecules and coagulation factor X in adenovirus 5-mediated gene transfer in pancreatic cancer cells. Cancer Gene Ther. 2011;18: 478–488. doi: 10.1038/cgt.2011.17 . 21566668

[12] Mizejewski GJ. Role of integrins in cancer: survey of expression patterns. Proc Soc Exp Biol Med. 1999;222: 124–138. doi: 10.1177/153537029922200203 . 10564536

[13] Hosotani R, Kawaguchi M, Masui T, Koshiba T, Ida J, Fujimoto K, et al. Expression of integrin alphaVbeta3 in pancreatic carcinoma: relation to MMP-2 activation and lymph node metastasis. Pancreas. 2002;25: e30–e35. doi: 10.1097/00006676-200208000-00021 . 12142752

[14] Kitahashi T, Yoshimoto M, Imai T. Novel immunohistochemical marker, integrin α_V_β_3_, for BOP-induced early lesions in hamster pancreatic ductal carcinogenesis. Oncol Lett. 2011;2: 229–234. Available from: ISI: 000287794100007. doi: 10.3892/ol.2011.252 22866069 PMC3410593

[15] Yoshimoto M, Hayakawa T, Mutoh M, Imai T, Tsuda K, Kimura S, et al. In vivo SPECT Imaging with ^111^In-DOTA-c(RGDfK) to detect early pancreatic cancer in a hamster pancreatic carcinogenesis model. J Nucl Med. 2012;53: 765–771. Epub 2012/04/13. doi: 10.2967/jnumed.111.099630 . 22496584

[16] Cai W, Chen X. Preparation of peptide-conjugated quantum dots for tumor vasculature-targeted imaging. Nat Protoc. 2008;3: 89–96. doi: 10.1038/nprot.2007.478 . 18193025

[17] Kimura S, Masunaga S, Harada T, Kawamura Y, Ueda S, Okuda K, et al. Synthesis and evaluation of cyclic RGD-boron cluster conjugates to develop tumor-selective boron carriers for boron neutron capture therapy. Bioorg Med Chem. 2011;19: 1721–1728. doi: 10.1016/j.bmc.2011.01.020 . 21315608

[18] Ogihara-Umeda I, Sasaki T, Kojima S, Nishigori H. Optimal radiolabeled liposomes for tumor imaging. J Nucl Med. 1996;37: 326–332. [Epub 1996/02/01]. . 8667071

[19] Kluza E, van der Schaft DW, Hautvast PA, Mulder WJ, Mayo KH, Griffioen AW, et al. Synergistic targeting of alphavbeta3 integrin and galectin-1 with heteromultivalent paramagnetic liposomes for combined MR imaging and treatment of angiogenesis. Nano Lett. 2010;10: 52–58. doi: 10.1021/nl902659g . 19968235

[20] Umeda IO, Tani K, Tsuda K, Kobayashi M, Ogata M, Kimura S, et al. High resolution SPECT imaging for visualization of intratumoral heterogeneity using a SPECT/CT scanner dedicated for small animal imaging. Ann Nucl Med. 2012;26: 67–76. doi: 10.1007/s12149-011-0542-7 . 21987284

[21] Thigpen JT, Aghajanian CA, Alberts DS, Campos SM, Gordon AN, Markman M, et al. Role of pegylated liposomal doxorubicin in ovarian cancer. Gynecol Oncol. 2005;96: 10–18. doi: 10.1016/j.ygyno.2004.09.046 . 15589573

[22] Wang-Gillam A, Hubner RA, Siveke JT, Von Hoff DD, Belanger B, de Jong FA, et al. NAPOLI-1 phase 3 study of liposomal irinotecan in metastatic pancreatic cancer: final overall survival analysis and characteristics of long-term survivors. Eur J Cancer. 2019;108: 78–87. Epub 20190114. doi: 10.1016/j.ejca.2018.12.007 . 30654298

[23] Accardo A, Mansi R, Salzano G, Morisco A, Aurilio M, Parisi A, et al. Bombesin peptide antagonist for target-selective delivery of liposomal doxorubicin on cancer cells. J Drug Target. 2013;21: 240–249. Epub 20121121. doi: 10.3109/1061186X.2012.741138 . 23167653

[24] Iwase Y, Maitani Y. Dual functional octreotide-modified liposomal irinotecan leads to high therapeutic efficacy for medullary thyroid carcinoma xenografts. Cancer Sci. 2012;103: 310–316. Epub 20111122. doi: 10.1111/j.1349-7006.2011.02128.x . 22017398

[25] Elamir A, Ajith S, Sawaftah NA, Abuwatfa W, Mukhopadhyay D, Paul V, et al. Ultrasound-triggered Herceptin liposomes for breast cancer therapy. Sci Rep. 2021;11: 7545. Epub 20210406. doi: 10.1038/s41598-021-86860-5 . 33824356 PMC8024284

[26] Huang Y, Huang Y, He J, Wang H, Luo Y, Li Y, et al. Pegylated immunoliposome-loaded endoglin single-chain antibody enhances anti-tumor capacity of porcine alpha1,3GT gene. Biomaterials. 2019;217: 119231. Epub 20190614. doi: 10.1016/j.biomaterials.2019.119231 . 31254933

[27] Kaeopookum P, Petrik M, Summer D, Klinger M, Zhai C, Rangger C, et al. Comparison of ^68^Ga-labeled RGD mono- and multimers based on a clickable siderophore-based scaffold. Nucl Med Biol. 2019;78–79: 1–10. Epub 20191025. doi: 10.1016/j.nucmedbio.2019.09.002 . 31678781

[28] Thumshirn G, Hersel U, Goodman SL, Kessler H. Multimeric cyclic RGD peptides as potential tools for tumor targeting: solid-phase peptide synthesis and chemoselective oxime ligation. Chemistry. 2003;9: 2717–2725. doi: 10.1002/chem.200204304 . 12772286

[29] Lasic DD, Martin FJ, Gabizon A, Huang SK, Papahadjopoulos D. Sterically stabilized liposomes: a hypothesis on the molecular origin of the extended circulation times. Biochim Biophys Acta. 1991;1070: 187–192. doi: 10.1016/0005-2736(91)90162-2 . 1751525

[30] Woodle MC, Matthay KK, Newman MS, Hidayat JE, Collins LR, Redemann C, et al. Versatility in lipid compositions showing prolonged circulation with sterically stabilized liposomes. Biochim Biophys Acta. 1992;1105: 193–200. doi: 10.1016/0005-2736(92)90194-q . 1586658

[31] Allen TM, Chonn A. Large unilamellar liposomes with low uptake into the reticuloendothelial system. FEBS Lett. 1987;223: 42–46. doi: 10.1016/0014-5793(87)80506-9 . 3666140

[32] Allen TM, Hansen C. Pharmacokinetics of stealth versus conventional liposomes: effect of dose. Biochim Biophys Acta. 1991;1068: 133–141. doi: 10.1016/0005-2736(91)90201-i . 1911826

[33] Yonenaga N, Kenjo E, Asai T, Tsuruta A, Shimizu K, Dewa T, et al. RGD-based active targeting of novel polycation liposomes bearing siRNA for cancer treatment. J Control Release. 2012;160: 177–181. Epub 20111013. doi: 10.1016/j.jconrel.2011.10.004 . 22019557

[34] Zhao H, Wang JC, Sun QS, Luo CL, Zhang Q. RGD-based strategies for improving antitumor activity of paclitaxel-loaded liposomes in nude mice xenografted with human ovarian cancer. J Drug Target. 2009;17: 10–18. doi: 10.1080/10611860802368966 . 19016068

[35] Yu Q, Qiu Y, Wang X, Tang J, Liu Y, Mei L, et al. Efficient siRNA transfer to knockdown a placenta specific lncRNA using RGD-modified nano-liposome: A new preeclampsia-like mouse model. Int J Pharm. 2018;546: 115–124. Epub 20180503. doi: 10.1016/j.ijpharm.2018.05.001 . 29729405

[36] Ren Y, Yuan B, Hou S, Sui Y, Yang T, Lv M, et al. Delivery of RGD-modified liposome as a targeted colorectal carcinoma therapy and its autophagy mechanism. J Drug Target. 2021;29: 863–874. Epub 20210215. doi: 10.1080/1061186X.2021.1882469 . 33507113

[37] Li W, Su B, Meng S, Ju L, Yan L, Ding Y, et al. RGD-targeted paramagnetic liposomes for early detection of tumor: in vitro and in vivo studies. Eur J Radiol. 2011;80: 598–606. Epub 20110212. doi: 10.1016/j.ejrad.2011.01.051 . 21316892

[38] Kato N, Takahashi M, Tsuji T, Ihara S, Brautigam M, Miyazawa T. Dose-dependency and rate of decay of efficacy of resovist on MR images in a rat cirrhotic liver model. Invest Radiol. 1999;34: 551–557. doi: 10.1097/00004424-199909000-00001 . 10485069

[39] Xiao L, Li J, Brougham DF, Fox EK, Feliu N, Bushmelev A, et al. Water-soluble superparamagnetic magnetite nanoparticles with biocompatible coating for enhanced magnetic resonance imaging. ACS Nano. 2011;5: 6315–6324. Epub 20110802. doi: 10.1021/nn201348s . 21790153

[40] Hyeon T, Lee SS, Park J, Chung Y, Na HB. Synthesis of highly crystalline and monodisperse maghemite nanocrystallites without a size-selection process. J Am Chem Soc. 2001;123: 12798–12801. doi: 10.1021/ja016812s . 11749537

